# A Magic Filter Filled with Waste Plastic Shavings, Loofah, and Iron Shavings for Wastewater Treatment

**DOI:** 10.3390/polym14071410

**Published:** 2022-03-30

**Authors:** Zengrui Pan, Jianlong Sheng, Chong Qiu, Hongtang Wei, Qianjin Yang, Jinbo Pan, Jun Li

**Affiliations:** 1 Key Laboratory of Microbial Technology for Industrial Pollution Control of Zhejiang Province, College of Environment, Zhejiang University of Technology, Hangzhou 310014, China; pzr19970701@163.com (Z.P.); sjl19970316@163.com (J.S.); 2 Zhejiang Shuanglin Environment Co., Ltd., Hangzhou 311100, China; qiuchong0409@hotmail.com (C.Q.); weihongtang@slhj5.wecom.work (H.W.); 15868118495@163.com (Q.Y.); panjinbo@slhj5.wecom.work (J.P.)

**Keywords:** filter, waste fillers, pollutants removal, wastewater treatment

## Abstract

Integrated sewage treatment equipment has been widely used, but the commonly used fillers for wastewater treatment are not suitable in rural areas due to their price and performance issues. In this study, an integrated magic filter filled with waste fillers was proposed and established for wastewater treatment. The filter was composed of functional modules and an equipment room, and the fillers in each module can be taken out separately and changed arbitrarily according to the needs of specific treatment conditions. The fillers used include waste plastic shavings, loofah, and waste iron shavings, generated during the processing of plastic, crop, and steel. At the same time, a 91 d experiment was performed for real wastewater treatment, and a satisfactory removal performance was obtained, with average removal rates of COD, TP, NH_4_^+^-N, TN, and SS being 83.3%, 89.6%, 93.8%, 74.7%, and 94.0%, respectively. Through microscope observation, a large number of microorganisms were attached to the surface of the fillers, which was conducive to the simultaneous removal of nitrogen and phosphorus. The micro-electrolysis of waste iron shavings can produce Fe^2+^ and Fe^3+^, which would combine with PO_4_^3−^ to form Fe_3_(PO_4_)_2_ and FePO_4_ precipitates, enhancing the removal of phosphorus. In addition, the filled fillers have an excellent physical filtering effect, which can reduce the effluent SS. The magic filter achieves both the recycling of wastes and the treatment of wastewater.

## 1. Introduction

Due to the advantages of a small footprint and short construction period, integrated sewage treatment equipment has become one of the main options for rural sewage treatment [1,2,3,4]. In practice, biological fillers are often added to the integrated equipment for wastewater treatment [5,6,7]. Commonly used fillers include ceramsite, quartz sand, activated carbon, polyvinyl chloride, etc., but high costs, being easy to plug, and poor film hanging performance are the main problems for practice application [8,9,10,11,12]. In this regard, the filler with low cost, a wide source, high efficiency, and simple operation is still limited for rural sewage treatment.

Waste plastic shavings are generated during the processing of plastic, and its recycling has always been a problem. Plastic recycling plants process about 30% of the material received, while the remaining 70% is disposed of in landfills [13,14]. However, it should be noted that waste plastic shavings have many advantages, such as high strength, stable chemical properties, and easy availability [15,16], which basically meet the conditions for being used as biological fillers. In addition, waste iron shavings are produced in the process of steel processing and utilization, which is an easily available scrap metal in the form of rolled flakes, with Fe^0^ as the main component. Previous studies have confirmed that waste iron shavings have an excellent effect on phosphorus removal [17,18]. Loofah, an agricultural waste, is featured by its multi-layered fibrous network structure, making it an ideal microbial carrier. Meanwhile, loofah will slowly release the carbon source during the reaction process, thereby saving costs [19,20,21].

To this end, an integrated magic filter filled with waste fillers was proposed and established for real wastewater treatment. The fillers used included waste plastic shavings, loofah, and waste iron shavings. The structure of fillers and sludge morphology were observed and analyzed, and the pollutants removal performance of the magic filter was investigated. This work presents a valuable effort in expanding the practical application of waste fillers in wastewater treatment.

## 2. Materials and Methods

### 2.1. Design Thought

The thought of a magic filter is inspired by the Rubik’s cube, which can be combined freely and installed modularly. The magic filter includes an equipment room and functional modules. The equipment room is mainly responsible for controlling the operation of the magic filter, such as influent flow distribution, aeration, sedimentation, water flow direction, reflux, and disinfection. The functional modules are filled with different fillers according to the process and needs. A box-type aquatic plant can be placed on the top layer of the filter, which has both landscape and ecological pollutant removal effects. The functional modules are connected by connecting pipes, and the connection methods include welding, flange connection, flexible connection with rubber parts, etc.

In a practical application, the number of functional modules can be set flexibly according to the site conditions. Filler installation and replacement steps are as follows: filler is added to the filler frame according to the process requirements firstly, and then the filler frame is placed into the functional module in sequence, and finally, the landscape plant is set on the top of the filter. When the replacement of filler is required, the filler can be taken out simultaneously by taking out the filler frame, which is convenient for use.

### 2.2. Reactor Setup and Operation

**Figure 1:**
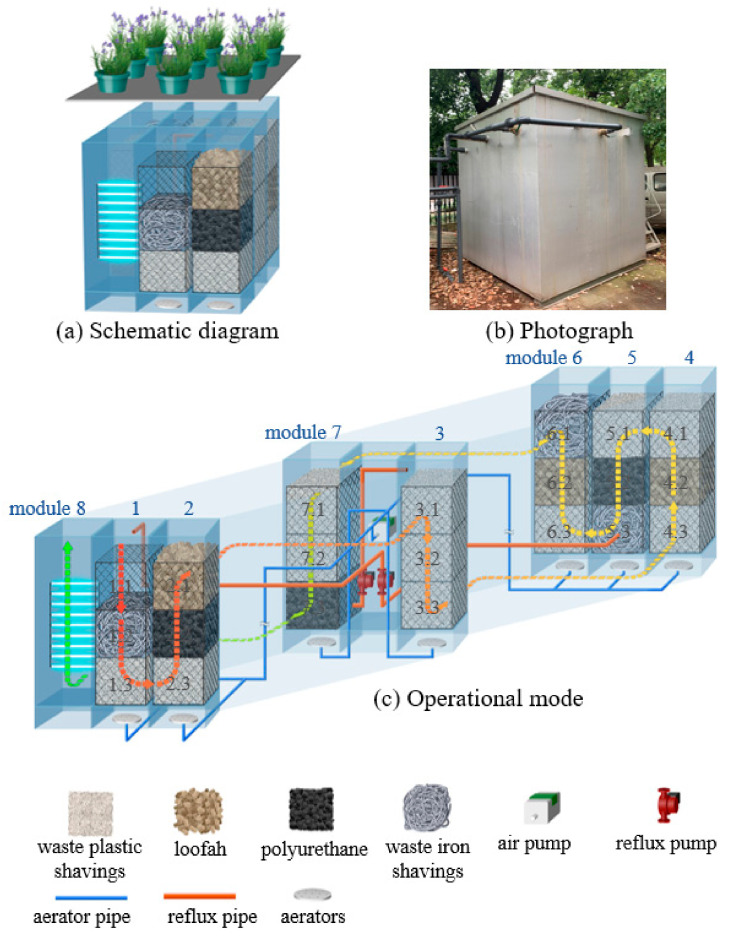
Schematic diagram, photograph, and operation mode of the magic filter.

According to the idea described above, a magic filter filled with various types of waste fillers was established and operated for real wastewater treatment, and a 91 d experiment was performed (Figure 1). The effective volume was 12 m^3^ (2.4 m × 2.4 m × 2.4 m), and the hydraulic retention time was 2 d. The reactor was composed of eight modules and an equipment room of the same size (0.8 m × 0.8 m × 2.4 m). The equipment room was located in the center of the reactor, module 8 was set for disinfection and sedimentation, and the remaining seven modules were filled with corresponding fillers according to processing requirements. A fine grille system (screen pore size of 1 cm), with the same size as the filler frame, was set in module 1.1 for eliminating the large-size particles in raw wastewater. Each module was loaded with three layers of filler frames (0.7 m × 0.7 m × 0.7 m), and each filler frame was equipped with brushes to prevent short flow. To isolate the aerator and support the filler frame, a support structure was provided at the bottom of the functional module. At the top of each module, boxed aquatic plants for landscape and biological deodorization were placed.

The following operational mode was determined via continuous optimization and debugging in the early operation (Figure 1c). The influent water was distributed to modules 1–3 using a three-stage non-uniform distribution, according to the 3:1:1 flow rate. There were two independent reflux pipelines connected by pipeline pumps in the equipment room, and the return paths were as follows: module 5 to module 1 and module 7 to module 3. In addition, air distribution pipes were installed in the equipment room and connected to aerators at the bottom of each module through an air pump and regulating valve. During operation, module 1 was set for consuming dissolved oxygen in raw wastewater through waste iron shavings, leaving module 2 in an anaerobic state. The dissolved oxygen of modules 3, 5, and 7 was controlled at 2–3 mg/L with an aerobic state, and modules 4 and 6 were controlled at 0.2–0.5 mg/L with an anoxic state.

### 2.3. Filler Selection and Characteristic

**Figure 2:**
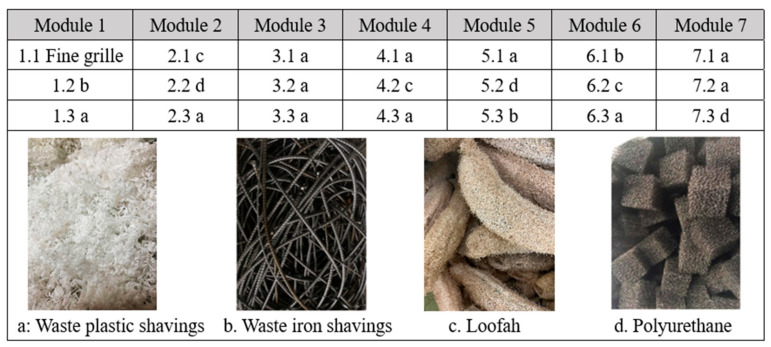
Filler composition of the magic filter.

The selection of filler should consider its physical filtration and biochemical effect, and from this, the following four fillers were identified: waste plastic shavings, waste iron shavings, loofah, and polyurethane (Figure 2). Among them, waste iron shavings are mainly used for phosphorus removal, and loofah is also a solid carbon source. Waste plastic shavings and polyurethane can be used as microbial attachment carriers. In addition, the above fillers also have a physical filtering effect.

Thereinto, the waste plastic shavings are obtained from a plastic product factory, in the shape of pleated waves and long strips, with a rough surface, and can produce large gaps between individuals after stacking. Furthermore, it has the advantages of high strength, excellent chemical stability, and easy to obtain, etc. The waste iron shavings are taken from a processing steel plant, with a spiral-shaped and 98.2% Fe content. The loofah comes from agricultural waste, which is an interwoven mesh of multi-layer filamentous fibers, with a long shuttle shape, lightweight, hard texture, slightly curved, thin at both ends, yellowish-white, etc. The polyurethane is purchased from an environmental protection enterprise, with a 99% open-pore rate, and has the advantages of a large specific surface area and easy biological adhesion.

The specific filler configuration is shown in Figure 2. Each filler basically fills the entire filler frame, and the mass of waste plastic shavings, waste iron shavings, loofah, and polyurethane for a single filler frame is about 20 kg, 200 kg, 5 kg, and 10 kg, respectively, and the filling rates are about 58.3 g/L, 583.1 g/L, 14.6 g/L, and 29.2 g/L, respectively.

### 2.4. Wastewater Characteristics and Seed Sludge

The wastewater comes from the domestic sewage of a company’s dormitory, which is collected in the septic tank and regulating tank before entering the magic filter. The raw wastewater quality varied greatly, and the main parameters of the real wastewater are as follows: COD (274.4 ± 100.0 mg/L), TN (121.9 ± 26.6 mg/L), NH_4_^+^-N (102.8 ± 16.1 mg/L), TP (7.9 ± 1.8 mg/L), SS (84.5 ± 38.6 mg/L). The reactor was inoculated with dewatered sludge obtained from a municipal WWTP in Hangzhou, China.

### 2.5. Analytical Methods

Wastewater samples were filtered through 0.45 μm filter paper before analysis. Parameters, such as COD, NH_4_^+^-N, TP, TN, and SS were measured using the standard methods [22]. Photographs of the fillers were taken using a stereo microscope (Olympus SZ61).

### 2.6. Statistical Analysis

An analysis method of the cumulative frequency with reference to the German ATV- DVWK-A 131E standard [23] was used to evaluate the reactor performance in pollutants removal.

## 3. Results and Discussion

### 3.1. Variation and Replacement of Fillers

**Figure 3:**
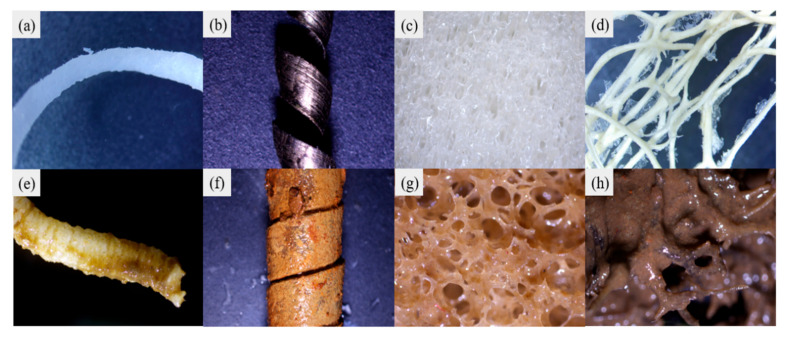
Images of the fillers before and after use (**a**–**h**).

The filler structure and sludge morphology were observed, as shown in Figure 3. The surface of the waste plastic shavings was wrinkled and wavy before use (Figure 3a), and a thick biofilm can be observed on the surface after use (Figure 3e), indicating that waste plastic shavings have good bioadhesive properties. In addition, the surface of waste iron shavings was shiny and spiral before use (Figure 3b), but the surface was continuously corroded due to micro-electrolysis during operation. As a result, the surface of the waste iron shavings was rough, and iron deposition and sludge could be clearly observed after use (Figure 3f). Polyurethane was a commonly used filler, and it has a porous mesh structure that makes it easy to adhere to the organism. As shown in Figure 3g, a large number of microorganisms were attached to the polyurethane from inside to outside after use. Loofah had a rough surface and porous structure, which was easily attached to by microorganisms (Figure 3d,h). In addition, carbon sources would be released slowly during operation to save carbon source input costs.

With the progress of the reaction, the cellulose, and other components in the loofah were continuously decomposed, so the carbon sources released gradually decreased and the structure gradually collapsed. Similarly, the surface of waste iron shavings would be gradually corroded under the effect of micro-electrolysis. It is time to replace waste iron shavings and loofah when the phosphorus and nitrogen removal performance is poor [18]. The replacement cycle of loofah and waste iron shavings is about 80 d in this study, while waste plastic shavings and polyurethane generally do not need to be replaced due to high wear resistance and strength [15,16,24,25].

### 3.2. Pollutants Removal

**Figure 4:**
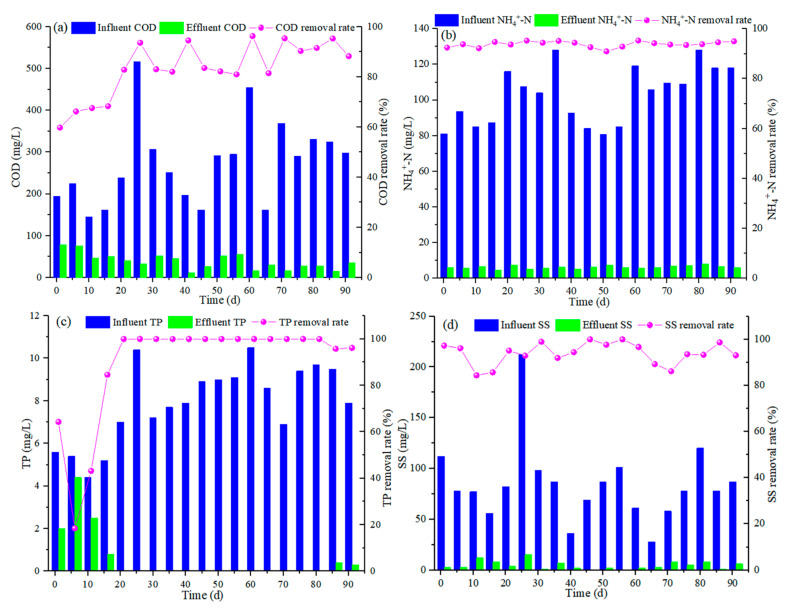
Variation of (**a**) COD; (**b**) NH_4_^+^-N; (**c**) TP; (**d**) SS.

Figure 4 depicts COD, TP, NH_4_^+^-N, and SS removal performance profiles of the reactor. Since this study treated real wastewater, the influent COD fluctuated significantly between 145 and 516 mg/L, resulting in a certain fluctuation of the effluent. During the first 16 d, the COD removal rate was 59.8–68.3%. With the continuous and stable operation of the reactor, the effluent COD concentrations were basically below 50 mg/L, and the removal rate of COD was 82.0–96.3%.

During the initial stage of operation, the removal rate of phosphorus fluctuates between 18.5% and 64.3% due to the release of a large amount of phosphorus from the seed sludge. With the reactor reaching the stable operation stage, the TP concentrations in the final effluent showed a significant decrease from 8.43 to 0.09 mg/L, with an average removal rate of 98.5%. It was speculated that the excellent removal rate of TP was related to the addition of waste iron shavings, which was confirmed by previous studies [17,18].

Moreover, the effluent NH_4_^+^-N concentrations (6.2 ± 0.8 mg/L) were relatively stable during the entire experimental period, with a wonderful removal rate of 93.8%, which showed that the filling fillers have an excellent removal performance on real wastewater. Despite the high TN concentrations (121.9 ± 26.6 mg/L) in the influent, the removal rate still reached 74.7%, which is related to the operation conditions, such as influent flow distribution, the addition of loofah, and the reflux of nitrifying liquid. In addition, the fillers used can effectively avoid the excessive wash-out of biomass at the initial stage of operation because of the good physical filtration performance. Thus, a stable and high removal efficiency of NH_4_^+^-N and TN can be maintained during the experimental period.

The influent SS fluctuated significantly during operation, with a maximum of 120 mg/L and a minimum of 36 mg/L, and an average of 84.5 mg/L. During the entire operation, the effluent SS remained stable below 15 mg/L, and the average removal rate was 94.0%. The low effluent SS was related to the fillers used in the reactor, particularly waste plastic shavings and polyurethane, which could effectively trap impurities and macromolecules in wastewater due to the microporous structure of the filler and the biological effect of surface attachment.

Due to the large variation of pollutant concentration in the influent water, the water quality data was statistically analyzed using the cumulative frequency method with reference to the German ATV- DVWK-A 131E standard [23], and the removal rate of COD, TN, NH_4_^+^-N, TP, and SS could be stabilized at 84.6%, 65%, 91.5%, 89.5%, and 92.0%, respectively. The above analysis shows that the reactor has a reliable and robust performance in pollutants removal.

### 3.3. Proposed Hypothesis of Pollutants Removal Path

As mentioned above, excellent removal performance was obtained, which is related to the operation mode and the fillers configuration of the reactor. The three-stage non-uniform distribution, and simultaneously two independent reflux pipelines (module 5–1 and module 7–3) can save the carbon sources of raw water, enhancing the removal of NO_3_^−^. Our previous study [18] has confirmed that the effect of iron shavings micro-electrolysis can produce Fe^2+^ and Fe^3+^, which would combine with PO_4_^3−^ to form Fe_3_(PO_4_)_2_ and FePO_4_ precipitates, enhancing the removal of phosphorus. In addition, the morphology of fillers after use showed that a large number of microorganisms are attached to the surface of the fillers, which was conducive to the formation of anaerobic–anoxic and aerobic environments and the simultaneous removal of nitrogen and phosphorus. At the same time, the filled fillers also have a physical filtering effect, which can retain iron precipitation and microorganisms, reducing the effluent SS.

## 4. Conclusions

In this study, an integrated magic filter filled with waste fillers was proposed and established. The filter consists of an equipment room and functional modules. The equipment room is mainly for controlling the operation of each module, and the functional modules can be filled with different fillers according to the process and needs. In addition, the reliable, and robust removal performance was obtained for wastewater treatment, with average removal rates of COD, TP, NH_4_^+^-N, TN, and SS being 83.3%, 89.6%, 93.8%, 74.7%, and 94.0%, respectively. The magic filter can be freely combined and installed according to the site conditions and obtain better sewage treatment performance by filling different types of waste fillers. Thus, the magic filter achieves both the recycling of wastes and the treatment of wastewater.

## Data Availability

The data presented in this study are available on request from the corresponding author.
